# Different photosynthetic responses of haploid and diploid *Emiliania huxleyi* (Prymnesiophyceae) to high light and ultraviolet radiation

**DOI:** 10.1186/s40643-023-00660-5

**Published:** 2023-07-14

**Authors:** Zuoxi Ruan, Meifang Lu, Hongmin Lin, Shanwen Chen, Ping Li, Weizhou Chen, Huijuan Xu, Dajun Qiu

**Affiliations:** 1 STU-UNIVPM Joint Algal Research Center, Guangdong Provincial Key Laboratory of Marine Biotechnology, Marine Biology Institute, Shantou University, Shantou, 515063 Guangdong China; 2 CAS Key Laboratory of Renewable Energy, Guangzhou Institute of Energy Conversion, Chinese Academy of Sciences, Guangzhou, 510640 Guangdong China; 3 CAS Key Laboratory of Tropical Marine Bio-Resources and Ecology, South China Sea Institute of Oceanology, Chinese Academy of Sciences, Guangzhou, 510301 Guangdong China; 4 Southern Marine Science and Engineering Guangdong Laboratory (Guangzhou), Guangzhou, 511458 Guangdong China; 5 College of Pharmacy and Life Sciences, Jiujiang University, Jiujiang, 332005 Jiangxi China

**Keywords:** *Emiliania huxleyi*, Diploid phase, Haploid phase, Effective quantum yield, Ultraviolet radiation (UVR)

## Abstract

**Abstract:**

Solar radiation varies quantitatively and qualitatively while penetrating through the seawater column and thus is one of the most important environmental factors shaping the vertical distribution pattern of phytoplankton. The haploid and diploid life-cycle phases of coccolithophores might have different vertical distribution preferences. Therefore, the two phases respond differently to high solar photosynthetically active radiation (PAR, 400–700 nm) and ultraviolet radiation (UVR, 280–400 nm). To test this, the haploid and diploid *Emiliania huxleyi* were exposed to oversaturating irradiance. In the presence of PAR alone, the effective quantum yield was reduced by 10% more due to the higher damage rate of photosystem II in haploid cells than in diploid cells. The addition of UVR resulted in further inhibition of the quantum yield for both haploid and diploid cells in the first 25 min, partly because of the increased damage of photosystem II. Intriguingly, this UVR-induced inhibition of the haploid cells completely recovered half an hour later. This recovery was confirmed by the comparable maximum quantum yields, maximum relative electron transport rates and yields of the haploid cells treated with PAR and PAR + UVR. Our data indicated that photosynthesis of the haploid phase was more sensitive to high visible light than the diploid phase but resistant to UVR-induced inhibition, reflecting the ecological niches to which this species adapts.

**Graphical Abstract:**

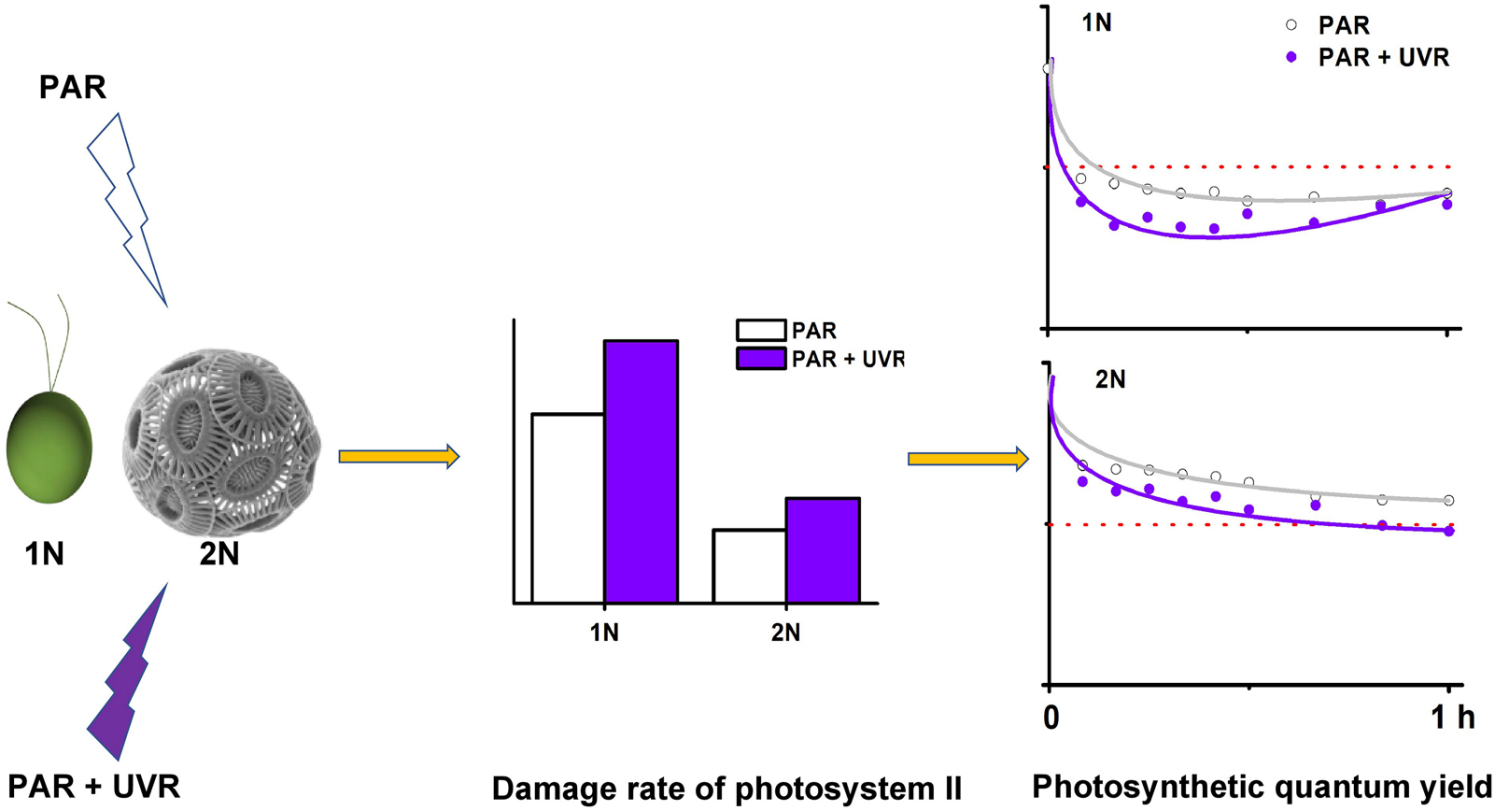

## Introduction

Coccolithophores are often covered with one or several layers of calcareous plates (i.e. coccoliths) around the cell surface. As one of the most important groups of marine phytoplankton, coccolithophores, together with other main calcifiers (e.g. foraminifera), account for almost half of the total production of CaCO_3_ in the pelagic zone (Balch et al. 2007; Brownlee et al. 2021). Therefore, they are of great importance in regulating the global biogeochemical carbon cycle.

*Emiliania huxleyi* is the most successful species of coccolithophores in the present-day ocean. It is frequently the dominant phytoplankton species in terms of cell number in surface seawater. More importantly, *E. huxleyi* forms extensive blooms with a large number of cells, and these blooms cover up to 1.4 × 10^6^ km^2^ of the world ocean annually (Holligan et al. 1993; Brown and Yoder 1994; Tyrrell and Merico 2004; Brownlee et al. 2021). Several environmental factors, including low silicate contents, high carbonate saturation state, etc., are considered facilitative for the development of blooms (Tyrrell and Merico 2004; Zondervan 2007; Pozdnyakov et al. 2021). However, high light conditions seem to be a crucial prerequisite for blooms (Tyrrell and Merico 2004; de Vries et al. 2020). At the sea surface or the water column surface, a high intensity of photosynthetically active radiation (PAR, 400–700 nm) is always accompanied by a high intensity of ultraviolet radiation (UVR, 280–400 nm), known as a stress factor damaging the protein and DNA of phytoplankton (Sinha and Häder 2002; Leunert et al. 2014; Haney et al. 2022). Depending on the scattering and absorption of seawater, UVR might penetrate through the water column to a depth of more than 20 m, where *E. huxleyi* is often observed and its blooms tend to occur (Nanninga and Tyrrell 1996; Falkowski and Raven 1997; Boelen et al. 1999; Frada et al. 2012; Jin et al. 2013; Pozdnyakov et al. 2021). *E. huxleyi* is thus expected to be exposed to UVR, especially if the vertical mixing of surface water is also considered (Jin et al. 2013).

The life cycle of *E. huxleyi* is typically composed of a diploid (2N) phase with coccolith-bearing or naked (without coccoliths) cells and a haploid (1N) phase with organic scale-bearing cells; both phases may propagate independently by mitosis (Green et al. 1996; Frada and Vardi 2017). The haploid and diploid phases of coccolithophores (e.g. *Calcidiscus leptoporus*, *Coccolithus pelagicus*) frequently concentrate in the upper photic zone during blooms (Frada et al. 2012; D’Amario et al. 2017). Although the diploid *E. huxleyi* can endure high light and does not show photoinhibition, even at 1000 µmol photons m^−2^ s^−1^ (Nanninga and Tyrrell 1996), it seems to be susceptible to UVR in terms of growth, photosynthesis, and calcification (Buma et al. 2000; van Rijssel and Buma 2002; Gao et al. 2009; Guan and Gao 2010a, b; Jin et al. 2022), which is similar to that observed for certain chlorophytes and diatoms (Lorenzo et al. 2019; Zang et al. 2022). Calcification, which is only found in diploid cells, may consume a significant part of the cell energy budget, mainly due to active ion transportation and coccolith polysaccharide production (Anning et al. 1996; Kayano and Shiraiwa 2009; Kayano et al. 2011; Monteiro et al. 2016; Vázquez et al. 2022). Such behaviour appears critical for the cells to avoid photodamage and maintain a relatively high photosynthesis performance, e.g. when cells are exposed to an abrupt increase in irradiance (Guan and Gao 2010a, b; Ramos et al. 2012; Xu et al. 2016). Coccoliths have also been shown to remove a considerable part of PAR and UVR, especially UVR-B, and they may also play a role in protecting the cell against high light and UVR (Gao et al. 2009; Guan and Gao 2010a, b; Xu et al. 2011, 2016). Although several studies have focused on the haploid phase of this species (mainly on its photosynthesis and interaction with the virus) (Houdan et al. 2005; Frada et al. 2008, 2012; Rokitta and Rost 2012; Mausz and Pohnert 2015; Frada and Vardi 2017; Alexander et al. 2020), little information is available on how UVR affects the haploid phase, which is critical for understanding the ecological niches and the succession of these two phases, especially when both phases coexist during bloom and even the prebloom period (Frada et al. 2012). Therefore, this work aimed to understand the different susceptibilities of photosynthesis of the life-cycle phases to high PAR and UVR.

## Materials and methods

### Culture conditions

The haploid strain RCC 1217 and the calcifying diploid strain PML B92/11 of *Emiliania huxleyi* were obtained from the Roscoff Culture Collection and originally from coastal waters of Bergen, Norway (Raunefjorden; 60°18.0′N, 05°15.0′E), respectively. The monospecific culture was maintained with an irradiance of 20.8 W m^−2^ (100 µmol photons m^−2^ s^−1^) and a 14-h light:10-h dark cycle at 20 °C in AMCONA artificial seawater media (see recepe in Fanesi et al. 2014). For the experiments, semicontinuous cultures were applied; the dilution rates were 0.40 d^−1^ for the haploid culture and 0.50 d^−1^ for the diploid culture based on their specific growth rates from batch cultures. The flasks were gently shaken twice daily during the light period to avoid cell sedimentation. Before the experiments, triplicate cultures were allowed to acclimate to the growth conditions for at least eight generations.

### Growth rate measurements

To minimize the background counts, sample aliquots from batch cultures were always bubbled with CO_2_ for 30 s to remove the coccoliths before cell counting. Cells were enumerated with a Z1 Coulter counter (Beckman Coulter Inc., Indianapolis, Indiana, USA). The specific growth rate (µ) was calculated using the following formula:

1 $$\mu \, = {\text{ ln}}\left( {{\text{C}}_{{2}} /{\text{ C}}_{{1}} } \right)/\left( {{\text{t}}_{{2}} {-}{\text{ t}}_{{1}} } \right).$$

C_1_ and C_2_ represent the cell concentrations at time t_1_ and time t_2_, respectively, both of which were at the exponential growth phase (Ruan and Giordano 2017).

### Cell size

Haploid or diploid cells in a haemocytometer were checked with an Axioplan 2 Imaging microscope (Zeiss Group, Oberkochen, Germany). Five to eight pictures were randomly taken, and cell diameters were measured by an Auxio Image System (Zeiss Group). The coccolith shell thickness was estimated from the size difference between intact cells (coccolith bearing) and naked cells (coccoliths being removed).

### Pigment quantification

Cells were collected by a Whatman GF/F glass fibre filter (General Electronic Company, Boston, Massachusetts, USA) and were placed in 7 ml of 90% acetone at 4 °C overnight. After centrifugation (5000×*g*, 10 min), the absorption spectrum of the supernatant (400–700 nm) was scanned with a Shimadzu UV-2501 spectrophotometer (Shimadzu Co., Kyoto, Japan), and the chlorophyll *a* and carotenoid contents were calculated according to the following equations (Strickland and Parsons 1972; Jeffrey and Humphrey 1975):

2 $${\text{chl a }}({\text{mg ml}}^{{ - {1}}} ) \, = \, \left( {{11}.{85 } \times {\text{ Abs}}_{{{663} - {665}}} {-}{ 1}.{54 } \times {\text{ Abs}}_{{{647}}} {-} \, 0.0{8 } \times {\text{ Abs}}_{{{63}0}} } \right),$$

3 $${\text{carotenoids }}\left( {{\text{mg ml}}^{{ - {1}}} } \right) \, = { 1}0.0 \, \times {\text{ Abs}}_{{{48}0}} ,$$

where Abs_663-665_, Abs_647_, Abs_630_, and Abs_480_ represent the absorption values at 663–665 nm, 647 nm, 630 nm, and 480 nm, respectively.

### High PAR and UVR exposure under a solar simulator

To assess the responses of different life-cycle phases to acute exposure to the high intensity of PAR and UVR, cultures were dispensed in 20 ml quartz tubes covered with Ultraphan 395 UV opaque foils (Digefra, Munich, Germany) or Ultraphan 295 UV-C cut-off foils (Digefra, Munich, Germany) to obtain the desired light treatments, i.e. PAR alone (irradiances above 395 nm) or PAR + UVR (irradiances above 295 nm). These tubes were incubated in a thermostated bath at growth temperature under a solar simulator (Honle UV Tech., Munich, Germany). The irradiance levels for PAR and UVR were approximately 83.3 W m^−2^ (400 µmol photons m^−2^ s^−1^) and 19.0 W m^−2^ UVR (18.4 W m^−2^ UV-A and 0.63 W m^−2^ UV-B), which is similar to the average level of daily intensity. The intensity of solar simulator radiation was recorded with an Eldonet radiometer (Realtime Computer Inc., Mohrendorf, Germany) according to Gao et al. (2009).

### Chlorophyll fluorescence measurements

Chlorophyll fluorescence was studied by a Water-PAM fluorometer (Heinz Walz, Pfullingen, Germany). Saturation pulse analysis was used to assess the photosystem yield (Schreiber et al. 1995). After 10 min of darkness, the minimal fluorescence (F_0_) was recorded under a weak measuring light (< 1 µmol photons m^−2^ s^−1^), low enough not to drive the electron flow; the maximum fluorescence (F_m_) was subsequently obtained following a saturated pulse (3260 µmol photons m^−2^ s^−1^, pulse width 0.8 s), and the maximum quantum yield was calculated as F_v_/F_m_ = (F_m_ − F_o_)/F_m_. Similarly, F_m_’ was excited by the saturated pulse (similar to the F_m_ measurement) after 20-s exposure to actinic light, which allowed the fluorescence to reach a steady state for all samples; before the application of the saturated pulse when actinic light exposure was going to end, the fluorescence intensity F_t_ was recorded. The effective quantum yield ΔF/F_m_’ was calculated as (F_m_’ − F_t_)/F_m_’. Non-photochemical quenching (NPQ) was determined based on the equation NPQ = (F_m_ − F_m_’)/F_m_’. To assess the responses of photosystem II of different life phases to high PAR and UVR stress, ΔF/F_m_’ and NPQ were tracked and determined every 5 min for the first 30 min and then every 10 min (Heraud and Beardall 2000; Xing et al. 2015). The repair and damage rates of photosystem II were analysed according to the following equation (Kok 1956; Heraud and Beardall 2000):

4 $${\text{P}}/{\text{P}}_{{{\text{initial}}}} = \, \left( {{\text{r }} + {\text{ k}}/{\text{exp }}\left( {\left( {{\text{k }} + {\text{ r}}} \right)/{\text{t}}} \right)} \right)/\left( {{\text{k }} + {\text{ r}}} \right).$$

P and P_initial_ represent the effective quantum yields ΔF/F_m_’ at time t and the onset of the experiment, respectively; r and k are the repair rate and the damage rate of photosystem II, respectively. To further confirm the effects of high light and UVR on the haploid and diploid photosystems, rapid light curves were recorded in the irradiance range of 0–716 µmol photons m^−2^ s^−1^ with 20 s exposure to each irradiance intensity. rETR, the relative electron transport rate of photosystem II, was estimated using the following equation:

5 $${\text{rETR }} = \Delta {\text{F}}/{\text{F}}_{{\text{m}}} \prime \times {\text{PFD }} \times \, a \times 0.5,$$

where PFD is the photon flux density, a is the absorption coefficient of chlorophyll *a*; and 0.5 represents the factor that accounts for energy partitioning between photosystem II and photosystem I. Since the absorption coefficient was not determined in this work, a constant value of 0.84 was used (Ruan et al. 2018). The rapid light curves were fitted with Origin 7.0 SR0 (OriginLab Co., Northampton, Massachusetts, USA.) according to the following model (Webb et al. 1974):

6 $${\text{P }} = {\text{ P}}_{{\text{m}}} \times \, \left( {{1 } - {\text{ exp}}\left( { - a \times {\text{ I}}/{\text{P}}_{{\text{m}}} } \right)} \right),$$

where P is the rETR at irradiance I; P_m_ is the maximum rETR and *a* is the maximum quantum efficiency of electron transport.

### Statistics

All data were acquired from three independent cultures and are expressed as the mean values with standard deviations. Homogeneity tests for variances were always assessed before further statistical analysis. The significance of variance was then checked by a two-tailed test or two-way ANOVA followed by LSD multiple comparison test by online statistical analysis SPSSAU (V2016-2023, QingSi Technology Ltd, Beijing, China). The significance level was always set at 95%.

## Results

**Fig. 1:**
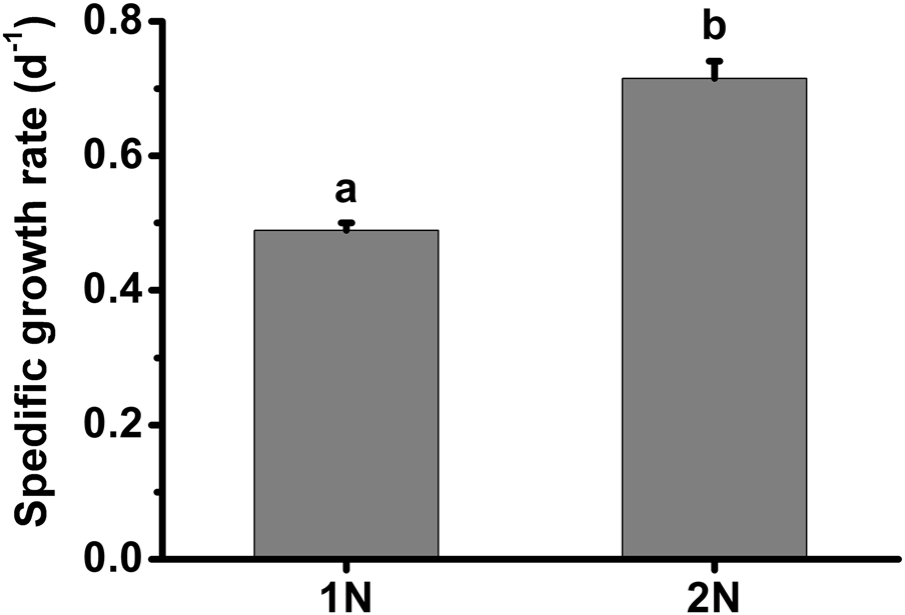
Growth rate of haploid (1N) and diploid (2N) *Emiliania huxleyi* acclimated to 100 µmol photons m^−2^ s^−1^ at 20 °C. Different superscripted letters indicate significantly different means (*P* < 0.05). Error bars denote standard deviations (*n* = 3)

**Fig. 2:**
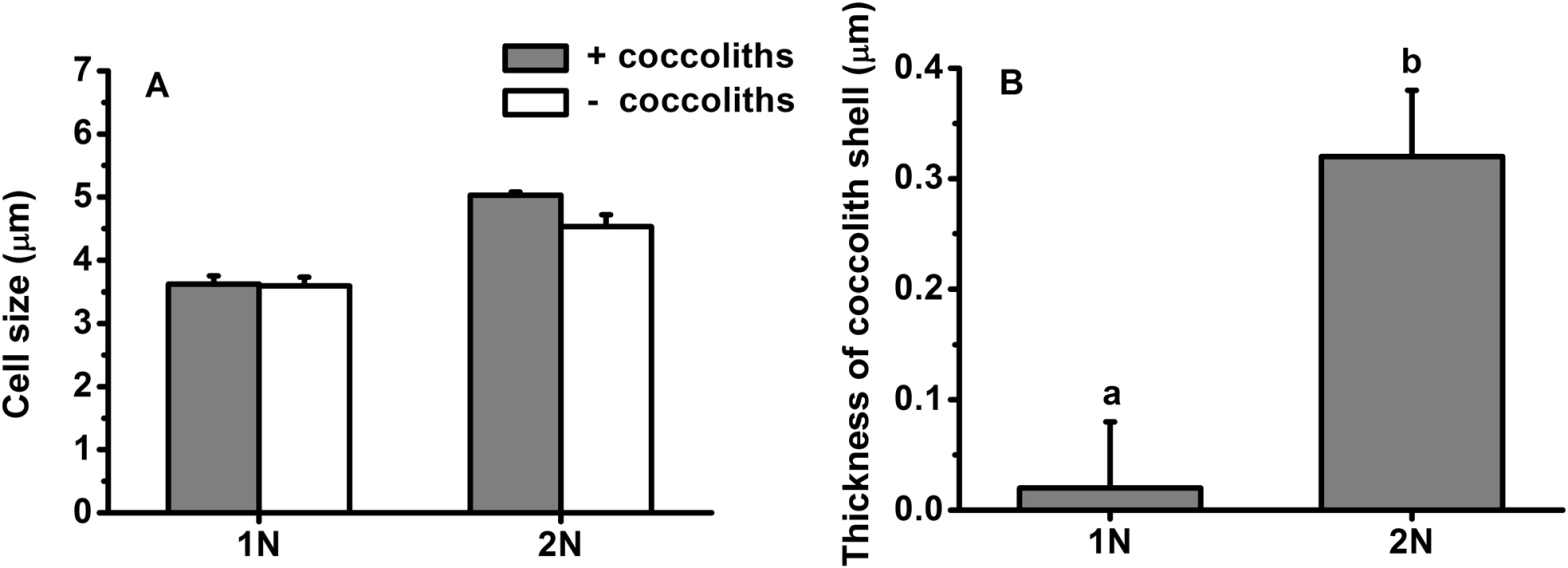
Cell size (**A**) and thickness of coccolith shell (**B**) of haploid (1N) and diploid (2N) *Emiliania huxleyi* acclimated to 100 µmol photons m^−2^ s^−1^ at 20 °C. + Coccoliths and − coccoliths denote the size of cells with and without (removed by bubbling CO_2_) coccolith shell. Different letters in superscript indicate significantly different means (*P* < 0.05). Error bars denote standard deviations (*n* = 3)

The specific growth rates of the haploid and diploid phases were 0.49 d^−1^ and 0.72 d^−1^, respectively (Fig. 1). Based on these growth rates, daily dilution rates at 0.40 d^−1^ and 0.50 d^−1^ were chosen for the haploid and diploid cultures. After at least eight generations of acclimation to the growth conditions, the cell sizes of various phases were significantly different (Fig. 2). The diploid cells (with coccoliths) were 5.0 µm in diameter, 39% larger than haploid cells (t-test, *P* = 0.00 < 0.01). The coccolith shell around the diploid cell surface was 0.3 µm thick (Fig. 2).

**Fig. 3:**
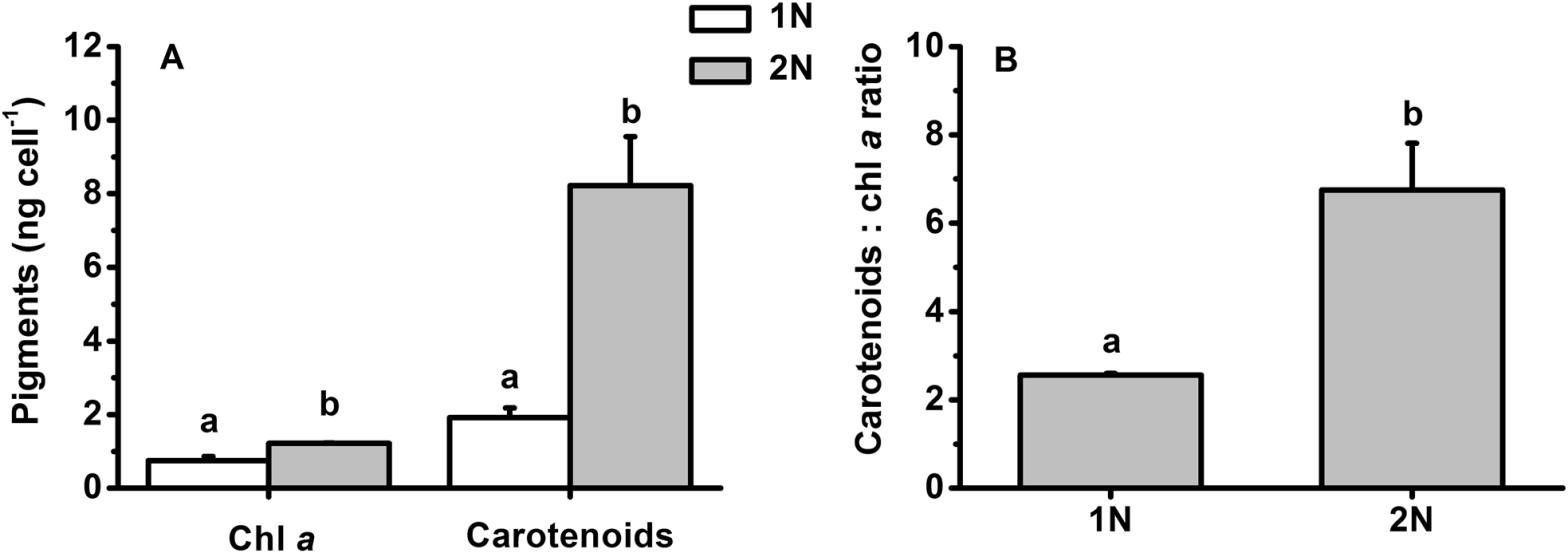
Pigments content (**A**) and carotenoid-to-chl *a* ratio (**B**) of haploid (1N) and diploid (2N) *Emiliania huxleyi* acclimated to 100 µmol photons m^−2^ s^−1^ at 20 °C. Different letters in the superscript indicate significantly different means between different life-cycle phases (*P* < 0.05). Error bars denote standard deviations (*n* = 3)

More pigments (62% for chlorophyll *a* and 328% for carotenoids) were observed in the diploid cells than in the haploid cells (Fig. 3A; t-test, *P* = 0.00 < 0.01); when the content of pigments was normalized to cell volume, no difference in chlorophyll *a* was found, yet carotenoids were still 182% more abundant. The carotenoids to chlorophyll *a* ratio of the diploid cells was higher (260%) compared with the haploid cells (Fig. 3B; t-test, *P* = 0.00 < 0.01).

**Fig. 4:**
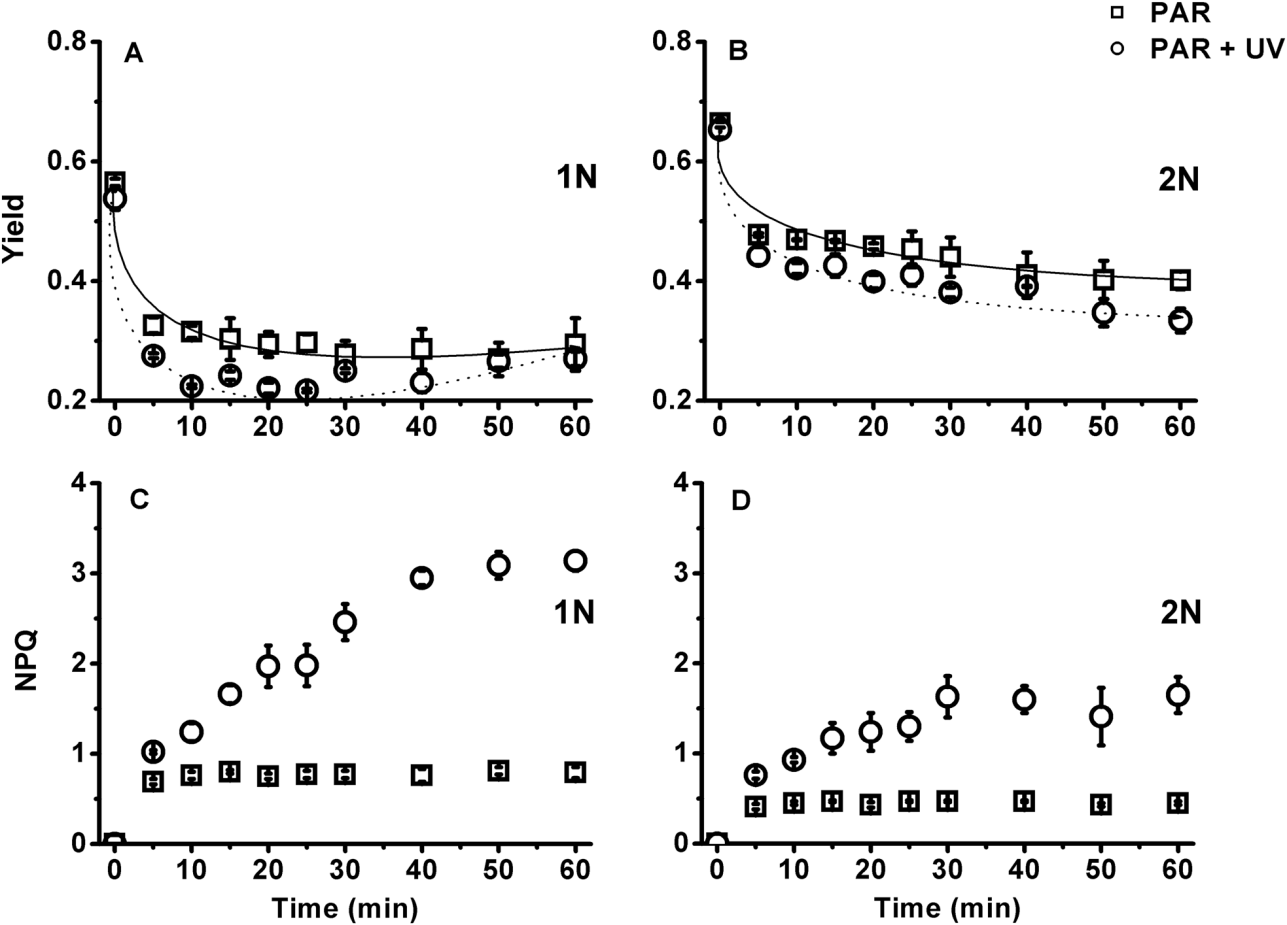
Dynamic changes in effective quantum yield (**A**, **B**) and nonphotochemical quenching (NPQ) (C, D) of haploid (1N) and diploid (2N) *Emiliania huxleyi* under a solar radiation simulator with 83.3 W m^−2^ PAR (400 µmol photons m^−2^ s^−1^) and 19.0 W m^−2^ UVR. Error bars denote standard deviations (*n* = 3)

When the cultures were exposed to PAR or PAR + UV, the effective quantum yield ΔF/F_m_’ decreased drastically in the first 5 min and reached its lowest values in the middle of exposure (25–30 min) for the haploid cells or at the end of the exposure (60 min) for the diploid cells (Fig. 4). Intriguingly, after 60 min of exposure, the effective yield of the haploid cells under PAR + UVR recovered from the lowest value of 0.22–0.28, a value comparable to that of the PAR treatment (Fig. 4A). Non-photochemical quenching NPQ was always higher in haploid cultures than in their counterparts, as is especially evident for those cultures exposed to PAR + UVR. NPQ reached its maximum value after 40 min for the haploid cultures and 30 min for the diploid cultures (Fig. 4C, D).

Although there was no significant difference in the repair rates (r) of photosystem II between different phases (F_(1,8)_ = 1.13, *P* = 0.32 > 0.05) or radiation treatments (F_(1,8)_ = 0.20, *P* = 0.67 > 0.05), the damage rates (k) of photosystem II in the diploid cells were approximately 59–60% lower than those of their haploid counterparts (F_(1,8)_ = 52.29, *P* = 0.00 < 0.05). The r/k ratios of the diploid cells were 1.5–1.7, whereas those of the haploid cells were lower than or close to 1.

**Fig. 5:**
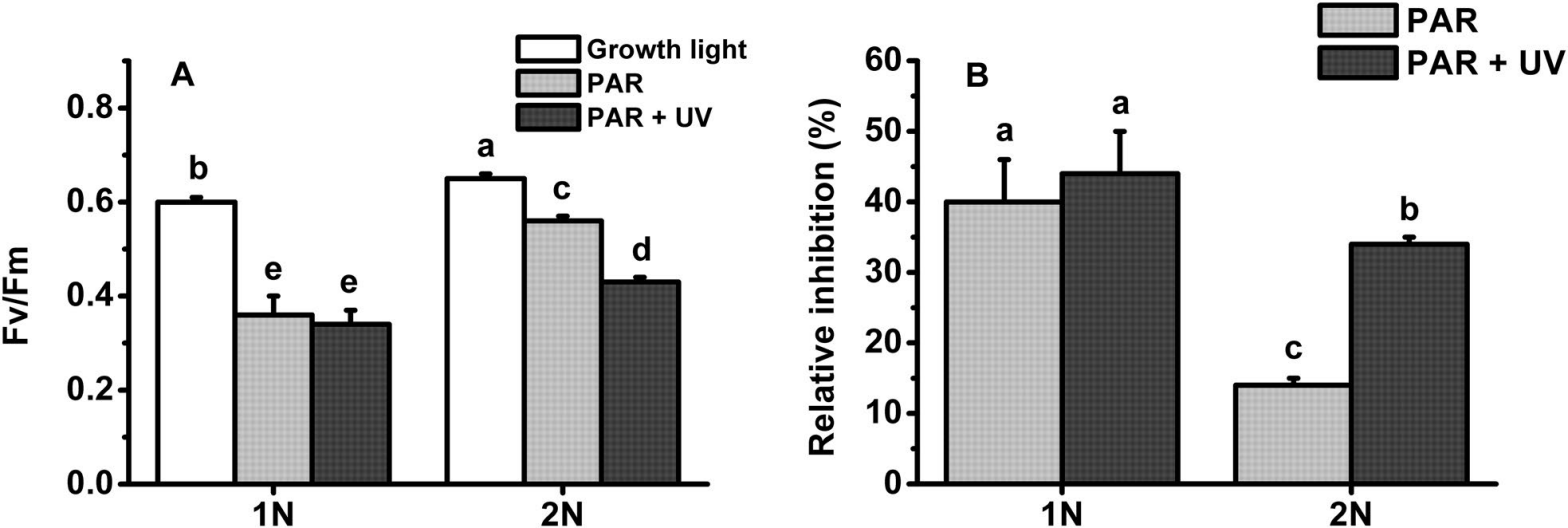
**A** Maximum quantum yield of haploid (1N) and diploid (2N) *Emiliania huxleyi* after acclimation to growth light or 1 h of acute exposure to PAR or PAR + UVR. **B** Relative inhibition of F_v_/F_m_ induced by PAR or PAR + UVR. The growth light, PAR and UVR were 20.8 W m^−2^ (100 µmol photons m^−2^ s^−1^), 83.3 W m^−2^ (400 µmol photons m^−2^ s^−1^) and 19.0 W m^−2^, respectively. Error bars denote standard deviations (*n* = 3). Different letters represent significantly different means between different life-cycle phases and different radiation treatments (*P* < 0.05)

To confirm the insensitivity of the haploid cells to UVR exposure, the maximum quantum yield (F_v_/F_m_) was determined after exposure to the solar simulator. F_v_/F_m_ between the haploid cells treated with PAR alone and with PAR + UVR was comparable (Fig. 5), and the relative inhibition rates (taking cultures maintained at growth light as a control) were 40% for the PAR treatments and 44% for the PAR + UVR treatments. However, the relative inhibition of F_v_/F_m_ caused by PAR + UVR was approximately 2.4-fold higher than by PAR alone in the diploid cells.

The photosynthesis parameters of the rapid light curves further confirmed the results of the previous experiment. The maximum relative electron transport rates (rETR_max_) of the haploid cells were significantly lower (34–39%) under the solar simulator than those acclimated to growth light (t-test, *P* = 0.00 < 0.05), although no significant difference in rETR_max_ was found between the PAR and PAR + UVR treatments (Table 2). No inhibition of rETR_max_ in the diploid cultures was caused by PAR alone, but PAR plus UVR resulted in 34% inhibition. The slope of the linear part of the curve (*a*) of the haploid cultures was more sensitive to high light exposure than that of the haploid cells: the relative inhibitions (compared with those maintained at growth light) were 16% (PAR) − 31% (PAR + UVR) for the haploid cultures and 3% (PAR) − 24% (PAR + UVR) for the diploid cultures (Table 2). The saturation irradiance (E_k_) was not affected by radiation (PAR or PAR + UVR; F_(2,12)_ = 3.23, *P* = 0.07 > 0.05), except for the haploid cultures exposed to PAR alone.

## Discussion

**Fig. 6:**
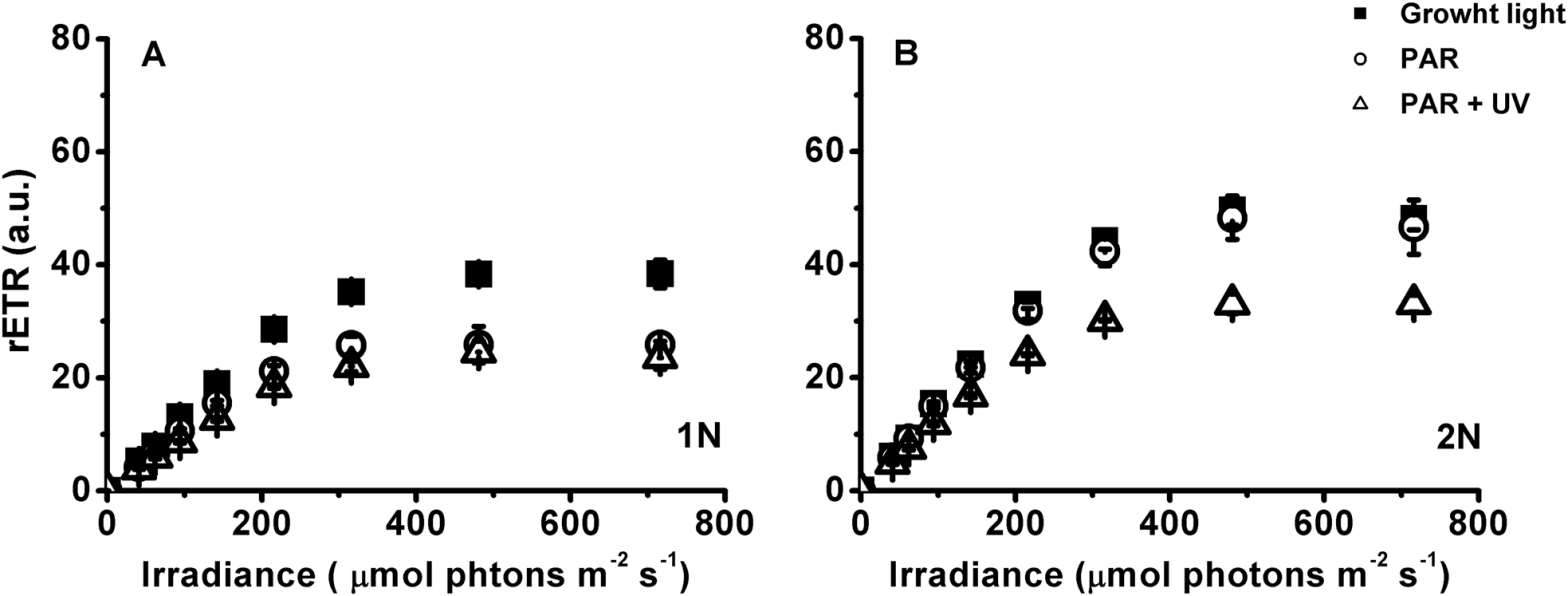
Rapid light curves of haploid (1N) and diploid (2N) *Emiliania huxleyi* after acclimation to growth light or 1 h of acute exposure to PAR or PAR + UVR. The growth light, PAR and UVR were 20.8 W m^−2^ (100 µmol photons m^−2^ s^−1^), 83.3 W m^−2^ (400 µmol photons m^−2^ s^−1^) and 19.0 W m^−2^, respectively. Error bars denote standard deviations (*n* = 3)

The haploid and diploid phases of *E. huxleyi* have marked morphological differences and share only half of the transcripts in common at the exponential stage, resulting in divergent physiologies in carbon and nutrient uptake and assimilation, energy budget, and biomass accumulation (Rokitta et al. 2011, 2012; Rokitta and Rost 2012; Alexander et al. 2020). It is, therefore, not surprising that the photosynthesis and the growth rate of these two phases were different (Fig. 1; Table 2), which were also reported previously (Houdan et al. 2005; Rokitta and Rost 2012; Mausz and Pohnert 2015). Moreover, the photosynthesis of haploid cells seemed to be more sensitive to the high PAR intensity but resistant to UVR-induced inhibition compared with the diploid counterparts (Figs. 4, 5, 6; Table 2).

Under supersaturating light, the diploid cells showed less photoinhibition than the haploid cells, which may be associated with a discrepancy in the capability of dissipating excess energy and subsequent production of reactive oxygen species that damage pigments and proteins and affect the structure and activity of photosystems (Foyer and Harbinson 1999; Shi et al. 2020). As one of the major sinks of cell energy, calcification is important in excess energy dissipation (e Ramos et al. 2012; Monteiro et al. 2016; Zhang and Gao 2021). The energy budget of calcification (including calcium transport, bicarbonate transport, polysaccharide generation, etc.) might account for up to 37% of the total photosynthetic energy (Monteiro et al. 2016). In particular, an abrupt increase in light intensity could cause an 11-fold increase in calcification with only a 5-fold increase in photosynthesis (e Ramos et al. 2012). That is, calcification may consume as much as 81% of the total photosynthesis energy, given that calcification and photosynthesis are operating at a comparable rate (Lorenzo et al. 2019). Calcification may therefore contribute to at least partly excess energy dissipation since the damage rate of photosystem II in the diploid cells was lower (Table 1). In addition, the calcareous shell formed by coccoliths also absorbs and/or attenuates a significant proportion of PAR (Gao et al. 2009). This may further alleviate high PAR stress. Although the coccosphere may remove up to 20% of PAR, the coccosphere in the present study is too thin (only 0.3 µm) and could reduce only 3% of solar irradiance based on our previous study (Fig. 2; Ruan et al. 2016). Therefore coccoliths *per se* in the present study might have a very limited contribution to photoprotection. In addition to calcification and coccolith shell, carotenoids often play a role in light harvesting or energy dissipation depending on the light availability (Goss and Lepetit 2015; Leverenz et al. 2015; Xi et al. 2022), and they were higher in the diploid cells (Fig. 3). Potentially, these calcified cells could be more active in dissipating energy via the xanthophyll cycle when subjected to supersaturated light. However, the NPQ values in our study did not seem to support this, as may be related to the fact that calcification was the main player in consuming excess energy. Thus, the less effective NPQ was actually an indication of less stress under high light, manifested by the lower damage rate in the diploid cells (Table 1), whereas the NPQ of the haploid cells could be the main path to disperse excess energy.

**Table 1:** Repair rate (r) and damage rate (k) of photosystem II obtained from effective quantum yield dynamics of haploid (1N) and diploid (2N) *Emiliania huxleyi* during 1 h of acute exposure to PAR or PAR + UVR under a solar radiation simulator according to Heraud and Beardall (2000)

	1N		2N	
	PAR	PAR + UVR	PAR	PAR + UVR
Repair rate (r)	0.19 ± 0.03^a^	0.20 ± 0.01^a^	0.12 ± 0.06^a^	0.14 ± 0.02^a^
Damage rate (k)	0.18 ± 0.03^b^	0.24 ± 0.01^a^	0.07 ± 0.02^c^	0.10 ± 0.02^c^
r/k ratio	1.06 ± 0.02^b^	0.84 ± 0.06^c^	1.67 ± 0.28^a^	1.48 ± 0.10^a^

PAR and UVR were 83.3 W m^−2^ (400 µmol photons m^−2^ s^−1^) and 19.0 W m^−2^, respectively. The values are shown as the mean ± standard deviation (*n* = 3). Different letters denote significantly different means between different life-cycle phases and radiation treatments (*P* < 0.05)

The presence of UVR led to a further decrease of quantum yield in the haploid cells compared with PAR alone. Intriguingly, this decrease completely recovered at the end of the exposure (Fig. 4), which was further confirmed by F_v_/F_m_ and other photosynthetic parameters (Fig. 6; Table 2). One of the reasons for this recovery may be attributed to the haploid cells being very active in repair and protein turnover (Table 1; Rokitta et al. 2011). Since no similar recovery was also observed under PAR alone, the enhanced repair in the haploid cells could be partly activated by UV-A/B. The UV-specific photoreceptor UV Resistance Locus 8 (UVR8) in the cytosol can be the candidate for this activation (Tilbrook et al. 2016). Upon UV exposure, UVR8 monomerizes to its active form and interacts with E3 ubiquitin ligase constitutively photomorphogenic 1 (COP1) in the nucleus to form the UVR8-COP1 complex (Tokutsu et al. 2021; Wang et al. 2022), which will change the expression of a serial of genes, e.g. D1 protein, to expedite the repair process and thus the recovery of quantum yield (Tilbrook et al. 2016; Giovagnetti and Ruban 2018). What’s more, two critical proteins LHCSR1 (photoprotective proteins LHC stress-related protein 1) and PsbS (photosystem II subunit S) contributing to NPQ can also be induced by UV exposure (Allorent et al. 2016), which may explain the sharp increase in NPQ of both haploidic and diploidic cells treated with UV. It is, however, noteworthy that a complete UVR8-COP1 signalling pathway has not been identified by far in the red lineage (e.g. diatom, coccolithophore) (Giovagnetti and Ruban 2018); whether this anterograde regulation also functions in coccolithophores is yet to be verified. Unlike haploid cells, the diploid cells were susceptible to UVR-induced inhibition: the damage rate of photosystem II increased by 43% (Fig. 6; Table 1). In addition, calcification is also very sensitive to UVR. In our previous study, calcification could be reduced by up to 44% under a similar level of UVR (Gao et al. 2009). Therefore, the reduction in calcification constrained excess energy dissipation and exacerbated the UVR stress that the diploid cells encountered.

**Table 2:** Parameters of the relative electron transport rate (rETR) versus irradiance curves of haploid (1N) and diploid (2N) *Emiliania huxleyi* after acclimation to growth light or 1 h of acute exposure to PAR or PAR + UVR

	1N			2N		
	Growth light	PAR	PAR + UVR	Growth light	PAR	PAR + UVR
rETR_max_	42.4 ± 2.6^b^	27.8 ± 1.9^d^	26.0 ± 2.5^d^	54.7 ± 2.8^a^	53.1 ± 5.8^a^	36.2 ± 2.2^c^
a	0.19 ± 0.01^b^	0.16 ± 0.01^c^	0.14 ± 0.01^d^	0.22 ± 0.00^a^	0.22 ± 0.01^a^	0.17 ± 0.01^c^
E_k_	221 ± 21^a^	173 ± 5^b^	199 ± 28^ab^	245 ± 10^a^	245 ± 21^a^	215 ± 24^a^

The growth light, PAR and UVR were 20.8 W m^−2^ (100 µmol photons m^−2^ s^−1^), 83.3 W m^−2^ (400 µmol photons m^−2^ s^−1^) and 19.0 W m^−2^, respectively. rETR_max_, maximum relative electron transport rate; a, slope of the light-limited portion of the rETR versus irradiance curve; E_k_ (µmol photons m^−2^ s^−1^), the irradiance at which energy becomes saturated for electron transport. The values are shown as the mean ± standard deviation (*n* = 3). Different letters denote significantly different means between different life-cycle phases and radiation treatments (*P* < 0.05)

Although the vertical distribution of the haploid phase of *E. huxleyi* is still unclear due to the lack of distinguishable coccolith, different photosynthetic responses of the haploid and diploid phases to acute exposure to high irradiance imply various ecological niches they occupy. The diploid phase tends to distribute even bloom in the surface water, where the light can be up to 1500 µmol photons m^−2^ s^−1^, because of the exceptional tolerance of high light. The haploid phase can recover from UVR-induced inhibition, as is important for this phase to immediately regain and maintain a relatively high photosynthesis in a variable environment of the surface seawater, e.g. during the process of the vertical mix. However, the photosynthesis of the haploid phase is in general more susceptible to high irradiance than the diploid phase, regardless of UVR. This implies that haploid *E. huxleyi* may thus tend to inhabit the relatively low part of the water column.

## Data Availability

The datasets used and/or analysed during the current study are available from the corresponding author on reasonable request.

## Abbreviations

1N: Haploid phase
2N: Diploid phase
F_v_/F_m_: Maximum quantum yield
NPQ: Non-photochemical quenching
PAR: Photosynthetically active radiation
UVR: Ultraviolet radiation
ΔF/F_m_’: Effective quantum yield
µ: Specific growth rate
